# Training-induced dynamics of accuracy and precision in human motor control

**DOI:** 10.1038/s41598-017-07078-y

**Published:** 2017-07-28

**Authors:** Abhishek Kumar, Yuto Tanaka, Anastasios Grigoriadis, Joannis Grigoriadis, Mats Trulsson, Peter Svensson

**Affiliations:** 1 Section of Oral Rehabilitation, Department of Dental Medicine, Karolinska Institutet, Huddinge, Sweden; 2 Department of Dentistry for Disability and Oral Health, Osaka Dental University Hospital, Osaka, Japan; 3 Section of Orofacial Pain and Jaw Function, Institute of Odontology and Oral Health, Aarhus University, Aarhus, Denmark; 4 SCON| Scandinavian Center for Orofacial Neurosciences, Huddinge, Sweden

## Abstract

The study investigated the dynamic changes in accuracy and precision during a simple oral and digital motor task involving a controlled and a ballistic force. Eighteen healthy participants participated in four experimental sessions during which they performed one hundred trials of targeting a controlled (low/high hold force) and a ballistic force during an oral and a digital motor task (OMT and DMT). Accuracy and precision across one hundred trials were calculated and subjected to segmented linear regression analysis. Repeated performance of controlled forces show a significant dynamic change in accuracy during initial stage of targeting high hold forces during OMT and a significant dynamic change in both accuracy and precision during final stage of targeting high hold forces during DMT. Repeated performance of ballistic force showed a significant dynamic change in both accuracy and precision during final stage of targeting high hold force forces during OMT and a significant dynamic change in accuracy during the initial stages of targeting high hold force during the DMT. The findings indicate a subtle degree of dissociation between accuracy and precision in terms of dynamic modulation of forces due to repeated performance of both OMT and DMT.

## Introduction

Fine manipulative skills are exceptional characteristics of the human species. The human manipulative superiority in comparison to other primates is probably due to intimate interactions between precise motor skills and higher cognitive functions^1^. Some very early observations in human motor behavior have suggested that human beings can very seldom replicate an exact movement in exactly same manner more than once. A simple example for this observation is to score an ace i.e., hole in one stroke while playing golf. One of the world’s greatest golfer could achieve this rare feat about eighteen times in his career but never twice consecutively. Hence, motor variability is one of the most common features in human movement performance and the variability in motor execution makes it virtually impossible to exactly repeat actions^2–4^.

The success of a motor task can be determined by either measuring the accuracy or the precision during the task performance. In the fields of science accuracy of a measurement system is determined by the degree of closeness of measurements of a quantity to that quantity’s true value. Whereas, the precision of a measurement system, is related to reproducibility and repeatability, i.e., the degree to which repeated measurements under unchanged conditions show the same results^5, 6^. It is suggested that although, the two words accuracy and precision are synonymously used in colloquial terms, they should deliberately be distinguished in the context of the scientific method.

It is suggested that the basic sensory and motor functions especially in relation to the precise manipulation of objects are similar in the trigeminally innervated jaw motor system and the spinally innervated hands and digits^7^. The perioral structures mainly the lips and the tongue along with the peripheral structures in the limbs particularly fingertips are well represented in the sensory and motor cortices. The mechanoreceptors in the skin of the hand and the orofacial region are found to be innervated by fast-conducting, large-diameter, myelinated a-beta axons, that respond vigorously to subtle mechanical deformation applied to their receptive fields^8^. However, despite these similarities studies have shown some differences in the cortical control and force execution between the trigeminally innervated masticatory muscles and spinally innervated hand muscles^9, 10^.

Therefore, the purpose of the present study was to investigate the dynamics of motor performance in terms of accuracy and precision due to repetition (training) of a similar oral and digital motor task. Further, we investigated if different target force levels would affect the force control mechanisms and particularly if they would play any role in modulating the dynamics of motor task performance. We hypothesized that repeated performance of the motor tasks would result in increase in the accuracy and precision of task performance. We also hypothesized that there would be no difference in the accuracy and precision of force control between trigeminally innervated jaw motor system and the spinally innervated hands/digits when similar motor tasks are performed.

## Materials and Methods

### Study participants

The study was conducted in accordance with the guidelines set forth in the Declaration of Helsinki II and informed consent was obtained from all participants prior to the start of the experiment. The study was approved by the regional ethical review board in Stockholm, Sweden. Eighteen healthy participants (Mean age: 27 ± 4 years, age range: 21–35 years, 5 women) were voluntarily recruited by advertising on a webpage (www.studentcannine.se) and were invited to participate in the study. The sample size calculation and power analysis was done on the basis of previous studies involving similar behavioural tasks^11, 12^. The present study was designed to detect a minimum difference in means of 30%. Power analysis indicated that 17–18 subjects would be sufficient to detect changes in force levels of 25–30% with an estimated variation of the outcome parameters corresponding to 25–30% and a risk of type I and II errors of 5% and 20%. The volunteers participating in the study were in good general health with no self-reports of functional or neurological problems related to biting, chewing or general dexterity. A simple temporomandibular disorder screening questionnaire ruled out the possibility of the participants suffering from any orofacial pain or temporomandibular disorder^13^. An intraoral examination by the examiner revealed that the participants were free from any ongoing or previous prosthetic or endodontic treatment and gross malocclusion of the anterior teeth including overjet/overbite.

### Armamentarium

**Figure 1:**
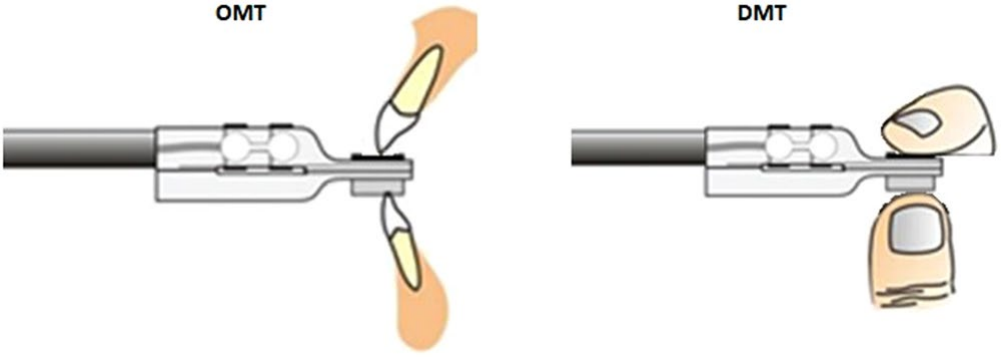
Schematic representation of an oral motor task (OMT) and digital motor task (DMT).

The force transducer used in the current experiment has been described in more detail in the previous studies^14–16^. Briefly, the experimental set up consisted of a custom made strain gauge based force transducer (Umeå University, Physiology Section, IMB, Umeå, Sweden) and a computer display monitor. The force transducer consisted of a lightweight metallic tube handle connected to two duralumin blocks terminating as rectangular plates (Fig. 1). The upper plate contained the force transducer for continuous measurement of forces applied to the upper plate with a recording frequency of 200 Hz. The force transducer was designed in such a way to ensured that the force measurement was independent of where the force was applied on the plate.

### Experimental paradigm

The volunteers were asked to participate in four experimental sessions scheduled on two different days (i.e., 2 sessions per day) separated by a minimum gap of one week. Each experimental session was of approximately forty five minutes duration. During the experimental session the participants were seated comfortably on an office chair with their hands resting on a table placed in front of them. On the table also rested a display monitor and a computer connected to a strain gauge based force transducer. The computer monitor displayed three target lines to demarcate a low controlled force (0.5N), a high controlled force (5N) and a ballistic force (10N) levels. During the experimental session the participants were asked to look and concentrate on the display monitor which displayed a moving waveform corresponding to the force applied on the force transducer. The task required the participants to continuously adjust the applied force to track the target line by biting/pinching on the force transducer.

### Behavioral task

Most oral and manual tasks in day to day life (for example; biting on a food morsel, pressing an electric switch or key board, playing a wind instrument etc.), either depend on the application of controlled forces or ballistic forces or a combination of both. Therefore, it was decided to study the level of force control during the behavioural task comprising of a controlled and ballistic force component. The application of the same force also allowed cross-comparisons in the level of accuracy and precision between the oral and digital motor systems. During the experimental session the participants were asked to perform ten series with ten trials of an oral and digital motor task (OMT and DMT) (Fig. 1). During the OMT the participants held the strain gauge based force transducer with their preferred hand and placed it on their lower central incisors. The motor tasks (OMT and DMT) comprised of a “steady” element called the controlled force characterized by low hold force levels and a “power” element called the ballistic force characterized by a fast, rapid ramp increase in force levels. The participants were asked to bite on the force transducer and target either a steady and controlled “low force” (i.e., 0.5N) or a steady and controlled “high force” (i.e., 5N) for about 3–5 seconds and thereafter rapidly increase the force to target a “ballistic force” (10N). Similarly, during the DMT the participants were asked to grasp the force transducer in between their thumb and index finger of their dominant hand and target either a steady controlled “low force” or a steady and controlled “high force” for about 3–5 seconds and a “ballistic force” as described for the OMT. The two controlled force levels (i.e., low and high force) are indicative of the tactile exploration (0.5N) and object manipulation force levels (5N), during object griping tasks^17–19^. While, the two controlled force levels correspond to the force levels within (0.5N) and beyond (5N) the mechanical sensibilities of the periodontal mechanoreceptors of anterior teeth during biting tasks^7, 20^. The participants performed ten series (with ten trials) each of OMT and DMT with a low controlled force and a high controlled force summing up to four hundred trails in total during the entire experiment. The order of the OMT and DMT along with controlled force levels were randomized.

### Data analysis

**Figure 2:**
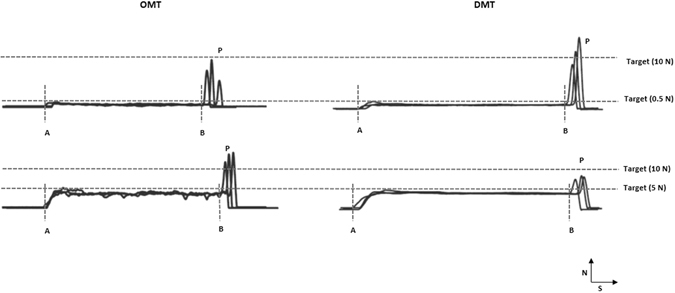
The typical force profiles obtained by superimposition of three trials of the task during the four experimental sessions. X-axis represents the time (S) and Y-axis represents force (N). Controlled force is the average force between the times intervals from the initial contact of the teeth/digits with the force transducer (**A**) until onset of the ballistic force (**B**). Ballistic force (P) is the maximum force prior to the moment after which the decline in force indicated by a rapid ramp decrease in the force occurred.

The typical force profiles obtained by superimposition of three trials of the task during the four experimental sessions are shown in Fig. 2. The force profile as a function of time was recorded and analyzed using an office computer at a recording frequency of 800 Hz and with customized software (WinZoom; Umeå University, Physiology Section, IMB, Umeå, Sweden). The specific points of interest during each individual trial were identified by the software and manually checked and corrected for any irregularities. The controlled force was defined as the average force between the times intervals from the initial contact of the teeth/digits with the force transducer (A) until onset of the ballistic force (B). The onset of the ballistic force was defined as the point at which the force rate exceeded 5N/s, the minimum rate of increase that could be reliably detected in a single trials^12, 16, 21–23^. The ballistic force was defined as the maximum force (P) prior to the moment after which the decline in force indicated by a rapid ramp decrease in the force occurred. Ballistic force was also the highest force during an individual trial.

### Statistics

**Figure 3:**
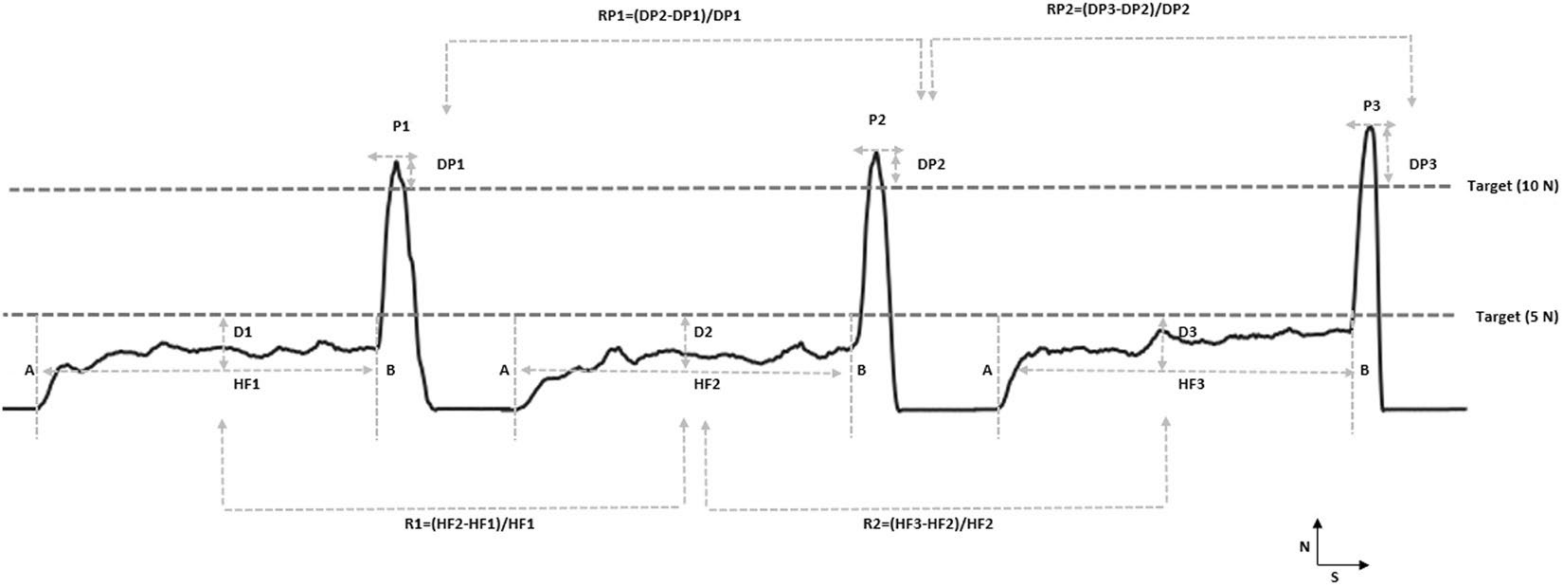
Examples of force profiles obtained from three consecutive trials of the behavioural task. X-axis represents the time (S) and Y-axis represents force (N). Controlled force (HF1, HF2, HF3) was defined as the average force between the times intervals from the initial contact of the teeth/digits (**A**) with the force transducer until onset of the ballistic force (**B**). The onset of the ballistic force was defined as the point at which the force rate exceeded 5N/s. The ballistic force was defined as the maximum force (P1, P2, P3) prior to the moment after which the decline in force indicated by a rapid ramp decrease in the force occurred. Accuracy: D1, D2, D3: deviation from the controlled force target (5N) and DP1, DP2, DP3: deviation from the peak force target (10N). Precision: R1, R2: relative change between trials for hold force and RP1, RP2: relative changes between trials for peak force.

Accuracy is a qualitative term that refers to whether there is an agreement between a measurement made on an object and its true (target or reference) value. Therefore, the accuracy of the task performance in the present study was assessed by calculating the percentage deviation of the forces (controlled force (i.e., D1, D2, D3) or ballistic force (i.e., DP1, DP2, DP3); see Fig. 3) from the target force during each trial for each condition. Whereas, precision which is described as the repeatability, or reproducibility of the measurement was determined in the present study by calculating the percentage relative change between the preceding trial to its subsequent trial (HF2-HF1/HF1 × 100) over one hundred trials, during each of the four conditions (see Fig. 3).

The dynamics of accuracy and precision across the four conditions in the present study are explained in terms of the tendency of the trend lines obtained by stepwise linear regression analysis. The waveform obtained by pooling all the hundred trials across the ten series were processed by smoothing the waveform by a moving mean of five consecutive trials. A linear regression analysis was done and analyzed for model fit. A stepwise/segmented linear regression analysis ensured the best fit. The two slopes of the regression lines were obtained from each participant during each session and each of the two slopes were compared to a horizontal line (i.e., slope = 0.00) with Wilcoxon Signed Ranks Test.The accuracy (deviation from the target) and the precision (relative changes between trials) were calculated for each trial and the mean of ten subsequent trials represented the series mean. The data corresponding to the outcome parameters were checked for assumptions of normality and logarithmic transformations were done wherever necessary. The data obtained were then subjected to a three-way repeated measures ANOVA model. The factors in ANOVA were condition (2 levels; OMT and DMT), controlled force (2 levels; 0.5N and 5N) and series (10 levels; 1–10). All post-hoc tests were performed with Tukey Honestly Significant Difference test for multiple comparisons. A P value of ≤0.05 was considered significant in all analysis.

## Results

### Dynamics of motor performance

The slopes and the intercepts obtained by stepwise linear regression analysis for the controlled force (low/high) and ballistic force in all four conditions are presented in Tables 1 and 2. A decrease in deviation from the target is indicative of an increase in accuracy of task performance and is represented by a negative slope of the trend line. Likewise, decrease in relative changes between the trials is indicative of an increase in precision of task performance and is also represented by a negative slope of the trend line. The dynamic changes in the slopes of the trend lines was evaluated by comparing each slope from all four sessions to a horizontal line. An “initial” increase in accuracy or precision was represented by a negative “first” slope while, a “final” increase in accuracy or precision was represented by a negative “second” slope.

**Table 1:** Slopes and intersection of the of the trend lines obtained by stepwise linear regression analysis for controlled force.

		Accuracy			Precision		
		Slope 1	Slope 2	Intersection	Slope 1	Slope 2	Intersection
OMT	LOW	−0.267	−0.822	49	0.283	−0.027	47
	HIGH	−0.513*	0.004	39	−1.424	−0.018	38
DMT	LOW	−0.882	−0.073	43	−0.960	−0.084	29
	HIGH	−0.560	−0.166*	34	−0.063	0.583*	49

**Table 2:** Slopes and intersection of the of the trend lines obtained by stepwise linear regression analysis for ballistic force.

		Accuracy			Precision		
		Slope 1	Slope 2	Intersection	Slope 1	Slope 2	Intersection
OMT	LOW	2.953	0.245	50	−3.14	0.345	40
	HIGH	−1.427	−0.721*	36	−0.234	−0.119*	28
DMT	LOW	−0.846	1.294	36	−10.54*	−0.171	23
	HIGH	−1.857*	−0.541	33	0.108	−0.355*	47

Asterisk (*) Significant differences between slope and horizontal line.

### Controlled force

**Figure 4:**
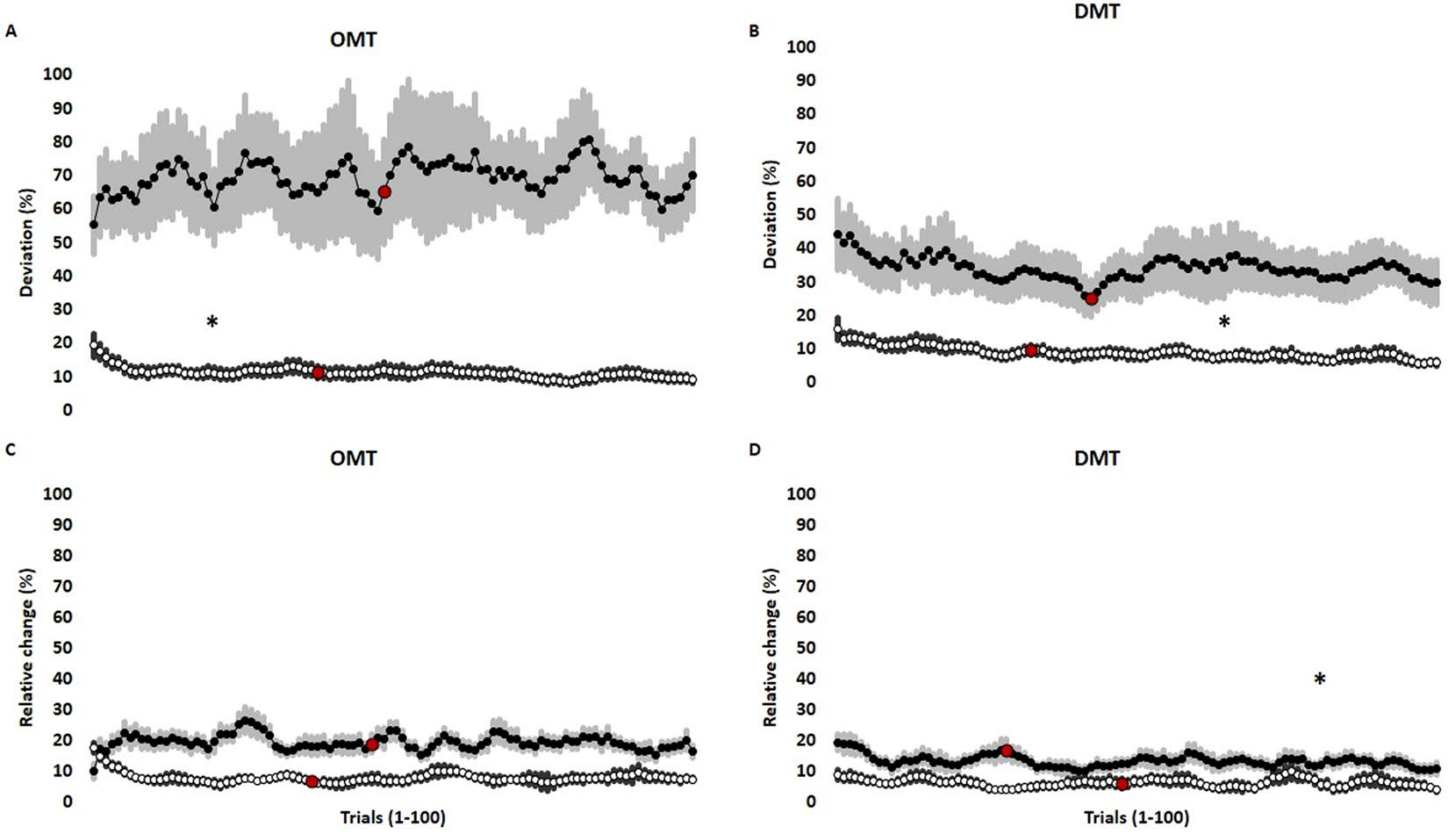
Mean and standard error of mean representing the dynamics of accuracy (**A**,**B**) and precision (**C**,**D**) of controlled forces during OMT (**A**,**C**) and DMT (**B**,**D**) with low hold force (black circles) and high hold force (white circles). The data represents the average of the one hundred trials from eighteen participants across each session. A stepwise/segmented linear regression analysis was done to study the dynamic changes. The two slopes (viz. initial stage and final stage) of the regression lines were obtained from each participant during each session and the two slopes were compared to a horizontal line. The intersection of the two slope are demarcated by red circles. The asterisk (*) denote significant differences between the high hold force slope and the horizontal line.

The controlled force during OMT and DMT was characterized by a steady and stable force corresponding to the target i.e., 0.5N or 5N. It was observed that there was an initial increase in the accuracy of task performance when targeting high controlled force during OMT (Wilcoxon Signed Ranks Test; P = 0.025). Whereas, there was a final increase in the accuracy of task performance when targeting high controlled force during DMT (Wilcoxon Signed Ranks Test; P = 0.002). There was also a final increase in the precision of task performance when targeting high controlled force during DMT (Wilcoxon Signed Ranks Test; P = 0.003) (Fig. 4).

### Ballistic force

**Figure 5:**
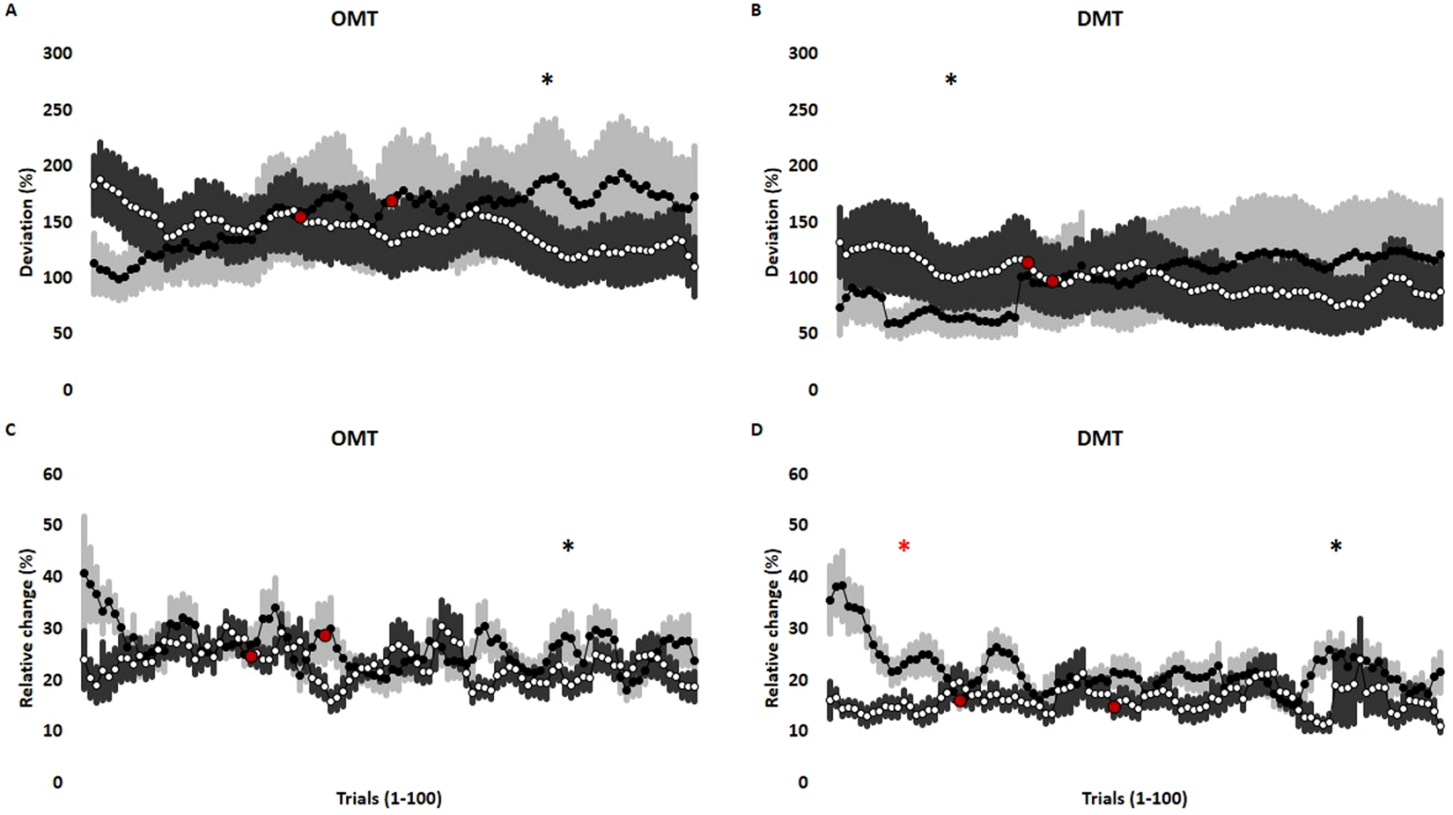
Mean and standard error of mean representing the dynamics of accuracy (**A**,**B**) and precision (**C**,**D**) of ballistic forces during OMT (**A**,**C**) and DMT (**B**,**D**) with low hold force (black circles) and high hold force (white circles). The data represents the average of the one hundred trials from eighteen participants across each session. A stepwise/segmented linear regression analysis was done to study the dynamics. The two slopes (viz. initial stage and final stage) of the regression lines were obtained from each participant during each session and each of the two slopes were compared to a flat line. The intersection of the two slope are demarcated by red circles. The black asterisks (*) denotes significant differences between the high hold force slope and the horizontal line. The red asterisks (*) denotes significant differences between the low hold force slope and the horizontal line.

The ballistic force during OMT and DMT was characterized by a very quick and short-lasting contraction of the jaw and hand muscles, respectively. The force profile obtained from such a ballistic movement showed a rapid ramp increase in force until the target of around (10N) was reached and a subsequent rapid ramp decrease in the force profile. It was observed that there was a final dynamic increase in the accuracy of task performance when targeting high controlled force during OMT (Wilcoxon Signed Ranks Test; P = 0.013). Whereas, there was an initial dynamic increase in the accuracy of task performance when targeting high controlled force during DMT (Wilcoxon Signed Ranks Test; P = 0.020). Further, there was a final dynamic increase in the precision of task performance when targeting high controlled force during OMT (Wilcoxon Signed Ranks Test; P = 0.044). There was also an initial and a final increase in precision when targeting low and high controlled force during DMT, respectively (Wilcoxon Signed Ranks Test; P = 0.006, P = 0.006) (Fig. 5).

### Accuracy in motor performance

#### Controlled force

The level of accuracy of the task performance across conditions, controlled force levels and series was assessed by calculating the deviation from the target force during a single trial and obtaining the series mean. The data thus obtained was subjected to a three-way repeated measures ANOVA model. The results of the ANOVAs and their interactions have been summarized in Table 3. The results revealed that there was a significant main effect of condition (F = 62.961, df = 15, P < 0.001), controlled force levels (F = 56.827, df = 15, P < 0.001) and series (F = 3.939, df = 135, P < 0.038). Post-hoc analysis of the result revealed that the deviation from the target was significantly greater during the OMT than during DMT (P < 0.001). The deviation was also significantly greater when targeting the low controlled force (i.e., 0.5N) than when targeting the high controlled force (i.e., 5N) (P < 0.001). Further, it was observed that the deviation from the target was lesser in the subsequent eighth and tenth series than the first and second series (P > 0.038).

**Table 3:** Summary of results of three-way repeated measures analysis of variance (ANOVA) for accuracy and precision of task performance.

	Accuracy		Precision	
	Controlled force	Ballistic force	Controlled force	Ballistic force
Condition	*	*	*	*
Control force level	*	NS	*	*
Series	*	NS	*	*
Condition × Control force level	*	NS	NS	*
Condition × Series	NS	NS	NS	NS
Control force level × Series	*	*	NS	*
Condition × Control force level × Series	NS	NS	NS	NS

*P < 0.05; NS: not significant statistically.

The results of the study also showed significant interactions between conditions (i.e., OMT or DMT) and controlled force levels (F = 6.730, df = 15, P = 0.020). Post-hoc analysis of the interaction revealed that the deviation from the target was greater when targeting low controlled force than when targeting either high controlled force levels during OMT or both low and high controlled force during DMT (P < 0.001). Also, the deviation from the target was greater when targeting low controlled force than when targeting high controlled force during both OMT and DMT (P < 0.001). There was also a significant interaction between controlled force levels and series (F = 3.892, df = 135, P < 0.001) with post-hoc tests showing significantly greater deviation in all the series when targeting low controlled force than targeting high controlled force (P < 0.001).

#### Ballistic force

There was a significant main effect of condition (F = 26.143, df = 14, P < 0.001). Post-hoc analysis of condition showed that the deviation from the target was greater during the OMT than during DMT (P < 0.001). Further, there was a significant interaction between the controlled force levels and series (F = 4.245, df = 126, P < 0.001). Post-hoc analysis of interactions revealed that the deviation from the ballistic force was smaller during the first series with low controlled force than the first series with high controlled force (P < 0.001).

### Precision in motor performance

#### Controlled force

The precision of task performance was assessed by calculating the relative change between the preceding trial to its subsequent trial and the data thus obtained was subjected to a three-way repeated measures ANOVA model. The results of the ANOVA revealed that there was a significant main effect of condition (F = 13.698, df = 15, P = 0.002) force levels (F = 85.347, df = 15, P < 0.001) and series (F = 2.716, df = 135, P = 0.006). Post-hoc analysis revealed that the relative changes between the trials was significantly higher during OMT than during DMT (P = 0.002) and significantly higher during low controlled force than high controlled force (P < 0.001). Further, the relative changes during the subsequent fourth, and seventh series was significantly lower than the first series (P < 0.033).

#### Ballistic force

There was a significant main effect of condition (F = 14.924, df = 14, P < 0.001), controlled force levels (F = 18.0877, df = 14, P < 0.001) and series (F = 4.589, df = 135, P < 0001) for the relative changes between trials for ballistic forces. Post-hoc analysis revealed that the relative changes between the trials was significantly higher during OMT than during DMT (P = 0.002) and significantly higher during low controlled force than high controlled force levels. Further, the relative changes between the trials was significantly higher in the first and third series than the subsequent eight and tenth series (P < 0.007).

The results also showed a significant interaction between condition and controlled force levels (F = 6.133, df = 14, P = 0.026). Post-hoc analysis of interaction showed that precision of ballistic force when targeting high controlled force during DMT was significantly lower than when targeting high controlled force both during OMT and DMT and low controlled force during DMT. Further, there was a significant interaction between controlled force levels and series (F = 2.126, df = 126, P = 003). Post-hoc analysis showed significantly higher precision during high controlled force during first, eighth, ninth and tenth series than the corresponding series with low controlled force.

## Discussion

Grasping and maintaining an object in a particular position requires the development of a precise and controlled force^24^. However, it is seldom possible that humans can maintain a constant stable clasp by applying an isometric force. It is often observed that individuals tend to either overshot or undershoot the target force initially during the clasp. However, in any motor task ones aim is to perform the task in hand accurately and in addition as precisely as possible; when the task is repeated. Previous research has addressed motor skill acquisition in terms of either the accuracy of task performance or trial-to-trial variability (measure of precision) in motor performance^25^. Nonetheless, due to the synonymous usage of these terms in colloquial terms often it is a challenge to distinguish their true interpretation in scientific studies. Therefore, the present experiment was designed to investigate the dynamics of accuracy and precision during a repeated oral and digital motor tasks involving a controlled and ballistic force. The results of the study are discussed in detail below.

### Dynamic modulation due to repeated performance of motor tasks

Motor skill acquisition during a skillful task is generally associated with learned behavior of force control and muscle activation which allows the individual to perform the task in hand, better than otherwise constrained. Skilled motor performance is suggested to be associated with improved accuracy and lower trial-to-trial variability compared with novice performance^26, 27^. The central nervous system dynamically calibrates the spatiotemporal representations of the object during a motor skill task despite the noisy, ever-changing environment^28^. The results of the present study shows a significant dynamic change in accuracy of controlled forces during the initial stage (first slope) of targeting high hold forces during OMT and a significant dynamic change in both accuracy and precision during the final stage (second slope) of targeting high hold forces during DMT. The results also show a significant dynamic change in both accuracy and precision of ballistic forces during the final stage of targeting high hold force forces during OMT and a significant dynamic change in accuracy during the initial stages of targeting high hold force during DMT. Further, there was also a significant dynamic change in precision of ballistic forces during the initial stages of targeting low hold force during OMT and final stages of targeting high hold force during DMT. These results indicate that there is a subtle degree of dynamic modulation of controlled and ballistic forces in terms of accuracy and precision due to repeated performance (training) of the OMT and DMT. Further, the results also indicate that training related dynamic modulations are associated with the magnitude of controlled force where high controlled forces (5N) are more likely to lead to dynamic changes (please see Tables 1 and 2).

The results also showed a significant dynamic modulation in accuracy of controlled force during the initial stages of targeting high hold force during OMT and a significant dynamic modulation in accuracy of ballistic forces during the initial stages of targeting high hold force during DMT; but no dynamic modulation in precision during these conditions. Further, it was also observed that there was a significant dynamic modulation in precision of ballistic forces during the initial stages of targeting low hold force and a significant dynamic modulation in precision of ballistic forces during the final stages of targeting high hold force, during DMT; yet no dynamic modulation in accuracy during these conditions. Therefore, these findings suggest a subtle degree of disassociation between accuracy and precision especially in relation to ballistic forces.

Skill acquisition and improved performance of a motor task with repeated practice results in decrease in variability of motor performance and increased representation of the trained muscles in the motor cortex^29, 30^. Previous studies have suggested that motor cortex is organized to coordinate movement sequences and that the development of new action patterns as a result of a novel skill acquisition is supported by changes in the topography of cortical movement representations^29, 31^. However, it is widely observed that motor variability is not completely eliminated despite expert performance^32–34^. In the present study despite a rigorous practice involving repetition of the same task for one hundred times the deviation from the target is still evident and there is no further improvement in performance. This evidence suggests that probably the ambiguous presence of variability is due to inherent “noise” in the nervous system^34^.

### Oral motor control vs. digital motor control

The OMT and DMT in the present experiment were similar in most aspects of force profiles produced and force vectors applied. Further, the oral mechanoreceptors have functional properties similar to the cutaneous mechoreceptors in the hand^7^. The results of the analysis (ANOVAs) showed that both the accuracy and the precision of task performance was significantly lower during OMT than during DMT. These findings indicate that despite similarities in relative force levels or force vectors, novelty of the behavioural task and properties of the peripheral receptors involved in the task; the functionality may still differ in terms of task performance between the two systems. We think that the differences in the motor performance can be due to the inherent differences between the systems. For example; previous studies have proposed that vision has been the modality most intensively investigated in the context of optimizing augmented feedback for motor learning^35^. It was suggested that the performance of the dexterous task requires precise dynamical control and would be dependent on the direction of fingertip force vectors with ample support from the visual, tactile, proprioceptive and multi joint inputs^35^. Therefore, lack of visual feedback could play an important role in downgrading motor performance in the trigeminal system compared to the spinal system in relation to motor learning. It has been argued that, due to the anatomic reasons, the projections on the trigeminal motoneurone pool from visual inputs are poor and the jaw movements cannot reach the same level of precision as limb movements where visual feedback is provided^24^. There are also biomechanical differences in muscle structure and fiber type between the oral and manual effectors^36^. Interestingly, it has also been reported that motor control of jaw muscles is less developed than that of the limb muscles during isometric contraction, when the target force level is presented by visual feedback^24^. The present study corroborates these findings where visual feedbacks were given during both tasks and yet there were differences in performances between the two systems.

Grasping of objects between the fingers is accomplished by a well-coordinated action where the two digits move towards each other and establish a stable contact with the object. The two movable digits would provide more flexibility for error compensation. Further, it is also suggested that the soft tissue covering the digital pads of the thumb and index finger would provide “cushioning” and also absorb any excessive forces during the object digit/finger contact. However, the jaw motor system is compromised in these respects since only the lower jaw is movable and lack of “cushioning” effects during the tooth object contact. It is also suggested that the tactile sensitivity of the orofacial tissues is higher than the other body areas which is attributed to high innervation density of mechoreceptors and high mechanical compliance of the orofacial tissues. High innervation density facilitates the likelihood of innervating some of the more sensitive afferents. Our results are in accordance with the previous studies where the authors report more variable force control with the oromotor structures than the fingers^36, 37^. Therefore, the findings from the present study suggests that perhaps the jaw movements cannot reach the same level of precision as limb movements despite visual feedback being provided^24^. It may be argued that in the present study DMT has perhaps more degrees-of-freedom than the OMT. Further, the oral task does not seem to be uncommon for the participants while, the digital task might have uncommon aspects. It is suggested that the differences between the two systems may be because unlike finger muscles, masticatory muscles do not need to be adapted to the task. Hence, future studies should be designed on the basis of uncontrolled manifold approach that has been developed to quantify statistically the extent to which variability of the motor elements leads to noise or error in performance versus reflecting the use of flexible patterns of coordination^38, 39^. Nevertheless, our present results imply that findings associated with studies from motor training in general cannot be extrapolated directly from the spinal to the trigeminal system and vice versa.

### Motor performance with different force levels

In the present study it was observed that both accuracy and precision of task performance was lesser when targeting low controlled force than when targeting high controlled force. Further, the interactions between the condition and force showed a lower accuracy of task performance when targeting low controlled force with the teeth during the OMT than high controlled force with the teeth or both low and high controlled force levels with the fingers. Therefore, it is suggested that force control tasks at higher force levels are less demanding than lower force levels in agreement with previous studies with finger force control tasks^40^. Previously, it has been suggested that motor unit firing rate variability and motor recruitment can be one of the causes for this observation. Motor units are recruited as per the “size principle” according to which the smallest motor units are recruited first, and then orderly recruitment of progressively larger units occur in order to increase force output^41^. It has also been reported that there is a positive correlation between force variability and relative motor unit firing variability at low force levels^34, 42^. Thus, increased higher motor unit firing variability at low force levels may result in decreased accuracy and precision at low force levels. Further, an increase in the number of motor units available decreases the force variability in continuous force production^43^. In the present study we have observed that there is greater variability at low hold force levels than at high hold force level. Several previous studies have also shown that accuracy and variability in force control is greatly compromised at low force levels than at high force levels^44, 45^. The present results also suggests that fine motor tasks involving isometric contraction with forces of small magnitude are perhaps difficult to be optimized due to the inherent noise in the nervous system. Nonetheless, variability (noise) should not be treated as “unwanted” and may rather be representing the exploration of motor command in space^46, 47^. We have previously observed that in the absence of target forces there is no optimization of jaw muscle activity and fine motor control in terms of decrease in variability of tasks performance, due to repetition of a similar standardized task^21^.

While both the accuracy and the precision of targeting ballistic force was smaller during both motor tasks (OMT and DMT); more robust effects were observed in terms of significant interactions in precision of task performance. It can also be noticed that the percent deviations are roughly twice during ballistic forces than during controlled forces. These results suggest that more pronounced deviations are evident during rapid force production tasks than force controlled tasks. It was suggested that rapid force production such as during ballistic movements predominantly relies on preplanned/feedforward/open-loop control, while continuous force maintenance such as in controlled forces primarily results from online feedback/closed-loop control^48, 49^. Production of ballistic forces involves discrete brief impulses to reach a given target and is dependent on high rate of force production and minimal use of sensory feedback^50^. Since the time scales are too short it does not allow neural signaling to either monitor or modify the movement once it has begun^51^. Hence higher variability is more evident during ballistic forces than during controlled forces.

## Conclusions

To summarize; the findings from the present study indicate a subtle degree of disassociation between accuracy and precision along with a subtle degree of dynamic modulation of forces in terms of accuracy and precision due to repeated performance of OMT and DMT. Further, we have observed a distinct difference in the mechanisms of force control in the trigeminally innervated jaw muscles compared to spinally innervated hand muscles when similar motor tasks were performed. The results also indicate that training related dynamic modulations are associated with the magnitude of controlled force where perhaps high controlled forces are more likely to show dynamic changes.

These results may be important because in recent decades, motor skill learning which relates to performance improvement beyond baseline has received less attention than motor adaptation, which relates to return to baseline performance despite external perturbation^25^. Isometric force control is one of the prominent paradigms that are currently used in motor control and aging research to explore different mechanisms underlying motor variability. A better understanding of these observed changes and their potential mechanisms will enable both researchers and clinicians to differentiate between aging and aging-related neuropathology such as Parkinson’s disease or implementation of neuroprosthetics. This study may serve as a foundation not only to examine differences in force control between healthy people and clinical patients, but also to explore learning or training effects of tactile exploration and manipulation. Improving force control by virtue of training we can expect improved functionality and increased therapeutic benefits in prosthodontic/prosthesis patients and further benefit practitioners in developing appropriate interventions to improve both oral and digital functional rehabilitation.
